# Microwave assisted drug delivery of titanium dioxide/rose Bengal conjugated chitosan nanoparticles for micro-photodynamic skin cancer treatment in vitro and in vivo

**DOI:** 10.1186/s12885-025-14285-8

**Published:** 2025-05-19

**Authors:** Samir Ali Abd El-Kaream, Nasser Ali Mohamed Hassan, Hassan Saleh Abdullatif Saleh, Mohamed Albahloul Salem Albahloul, Abdalla Mohamed Khedr, Sohier Mahmoud El-Kholey

**Affiliations:** 1 Applied Medical Chemistry Department, Medical Research Institute, Alexandria University, Alexandria, Egypt; 2 Medical Biophysics Department, Medical Research Institute, Alexandria University, Alexandria, Egypt; 3 Chemistry Department, Faculty of Science, Tanta University, Tanta, Egypt

**Keywords:** Skin cancer, Chitosan nanoparticle, Titanium dioxide, Rose Bengal, Micro-photodynamic

## Abstract

**Background:**

Micro-photodynamic therapy (MWPDT) combines photo-dynamic (PDT) and microwave-dynamic (MWDT) therapies with sensitizers, offers new avenues for cancer treatment. Despite the fact that novel sensitizers for MWPDT have been successfully synthesized, only a few are being employed effectively. The low tumor-targeting specificity, inability to transport sensitizer's deeper intratumorally, and deteriorating tumor microenvironment all restrict their anti-tumor efficacy. The current work was done aiming at microwave assisted drug delivery of titanium dioxide / rose Bengal conjugated chitosan nanoparticles (TiO_2_/RB@CSNP) for micro- photo-dynamic skin cancer (SKCA) treatment in vitro and in vivo as activated cancer treatment up-to-date modality.

**Materials and methods:**

The study was conducted in vitro on human SKCA cells (A-375) and the study protocol application groups in vivo on Swiss albino mice treated with 7,12-dimethylbenz[a]anthracene (DMBA)/croton oil only and were not received any treatment for inducing SKCA, and only after SKCA induction the study treatment protocol began, treatment was daily with TiO_2_/RB@CSNP as MWPDT sensitizer with or without exposure to laser (IRL) or microwave (MW) or a combination of them for 3 min for two weeks.

**Results:**

Revealed that CSNP can be employed as effective TiO_2_/RB delivery system that directly targets SKCA cells. Additionally TiO_2_/RB@CSNP is a promising MWPS for and when combined with MWPDT can be very effective in treatment of SKCA-A-375 in vitro (cell viability decreased in a dose-dependent basis, the cell cycle progression in G0/G1 was slowed down, and cell death was induced as evidenced by an increase in the population of Pre-G cells, an increase in early and late apoptosis and necrosis, and an increase in autophagic cell death) and DMBA/croton oil SKCA-induce mice in vivo (induced antiproliferative genes (caspase 3,9, p53, Bax, TNFalpha), suppressed antiapoptotic and antiangiogenic genes (Bcl2,VEGF respectively) effectively reducing the tumors growth and leading to cancer cell death as well as decreased oxidative stress (MDA), and ameliorated enzymatic and non-enzymatic antioxidants (SOD, GR, GPx, GST, CAT, GSH, TAC) as well as renal (urea, creatinine) and hepatic (ALT, AST) functions. This process could be attributed to MWPDT; microwave and/or photo-chemical TiO_2_/RB activation mechanism and antioxidant potential of non activated TiO_2_/RB as well.

**Conclusion:**

The results indicate that TiO_2_/RB@CSNP has great promise as an innovative, effective delivery system for selective localized treatment of skin cancer that is activated by MWPDT.

## Introduction

One of the most commonly diagnosed cancers groups worldwide is skin cancers (SKCAs) [1]. Global statistics from the SKCA Foundation indicate that one out of every three cancer diagnoses is a SKCA. Epidermis is made from keratinocytes, the predominant skin cells with melanocytes spread into the basal layer between the keratinocytes. The other cell types are rare and more present in the subepidermal layers and in the follicular structures. Also hypodermis is situated beneath the dermis and is a part of the skin. Any abnormality in this specific area of the skin could lead to a number of negative outcomes, such as the emergence of SKCA. SKCA is classified into two main categories: non-melanoma (NM) SkCA and melanoma (M) SKCA [1, 2]. Globally, NMSKCA is the most commonly diagnosed cancer kind, and MSKCA is associated with the highest number of deaths from cancer. In Egypt, 5% of all malignant tumors in the body were SKCAs. In Egypt, squamous cell carcinomas (SCC) account for 15% of cases, while melanomas account for 8% of cases. Basal cell carcinomas, or BCCs, account for 77% of all cases [1–4].

Nowadays, among other methods, SKCA is treated using radiation therapy, chemotherapy, cryosurgery, or surgical excision. There are benefits and drawbacks to each of the many treatment modalities, so selecting a course of action is never simple and is determined by a number of circumstances. Diligent research and the development of novel treatments for malignant tumors can improve the overall survival rate of SKCA patients. The use of therapeutic nano-formulation offers an alternate tactic to mitigate the harmful effects of synthetic materials [5, 6]. Furthermore, cutting-edge methods for treating malignant tumors are being actively studied and developed, which enhances patient survival in general. Micro-photo-dynamic therapy (MWPDT) is an alternate therapeutic approach for SKCA that use sensitizer in conjunction with microwave and photo-irradiation of the tumor to treat the condition. In order to initiate a variety of biological processes in the diseased area, MWPDT entails first giving a MWPS and then exposing the area to light and microwaves with the same absorbance wavelength as the MWPS to produce highly reactive oxygen species (ROS), such as peroxides (R–O–O^•^), hydroxyl radicals (^•^OH), and singlet oxygen (.^1^ O _2_), which cause irreversible damage to cancer cells. MWPDT inspired by photodynamic therapy (PDT), Micro-dynamic treatment (MWDT) has drawn interest as a potentially beneficial noninvasive treatment. PDT is ineffective in treating deeply embedded cancers due to its poor depth of light penetration. However, MWDT's main benefit over PDT is its ability to target soft tissue precisely and penetrate it up to tens of centimeters deep. MWDT thus resolves the main issue with PDT. In recent years, the use of MWPDT to treat a variety of tumors has grown in popularity, either by itself or in combination with other forms of treatment [7–11].

Titanium dioxide (TiO_2_) is one of the most basic materials in daily life that represents tremendous photo-catalyst activity. Recently, several functionalized biodegradable polymers were recently designed for TiO_2_ nanoparticles-based photo-thermal and photodynamic therapies. These photosensitizers can be modified by attaching dyes, targeting molecules, and drug molecules. Rose Bengal (4,5,6,7-tetrachloro-20,40,50,70-tetraiodofluorescein disodium, RB) is a dye belonging to the fluorescein family. This amphiphilic chemical molecule is already used in medical applications to test the activity of the liver and to diagnose corneal lesions. Subsequently, it had been discovered by researchers that RB stimulates the immune system, and making it possible to reduce the risk of certain cancers [12, 13].

Many sensitizers, including Rose Bengal (RB), and titanium dioxide (TiO_2_), have been found to respond to light irradiation in addition to being microwave activated, which may have implications for anticancer therapy through producing deadly singlet oxygen after being exposed to irradiation, which kills tumor cells. The microwave can also increase the amount of mechanical stress that causes cellular membrane breakage, which can further boost the microwave's potential to kill cells. However, RB showed a quick rate of clearance and minimal tumor buildup, which significantly constrained the use of RB in in vivo settings going forward. To overcome its shortcomings, sensitizers can be precisely delivered to the targeted tumor site using nanoparticle-based drug delivery systems because of their improved retention and penetration; appropriately sized nanoparticles should passively target and accumulate in tumors. Among these systems, a natural polysaccharide called chitosan, a derivative of chitin, has gained considerable attention as a biocompatible, biodegradable, and mucoadhesive material for creating nanoparticles. Chitosan nanoparticles provide several advantages, including improved stability, cellular uptake, solubility of anticancer drugs, modulation of release kinetics, and biodistribution. Additionally, chitosan nanoparticles can be modified on their surface with ligands or stimuli-responsive moieties to achieve targeted delivery to specific cancer cells or tissues. Furthermore, using MWPS's therapeutic substances in nano-formulations provides a substitute for the negative effects of synthetic medications [12–15].

Using biodegradable locally activated nanoparticles is another way to lessen the negative effects of traditional drugs. Additionally, state-of-the-art techniques for managing malignant tumors are continually being researched and developed, which improves overall patient survival. The anticancer properties of titanium dioxide/rose bengal chitosan based nanoparticles (TiO_2_/RB@CSNP) and their possible applications as MWPS for MWPDT have not been well explored. Therefore, the main objective of the present work is to deliver a novel investigation to evaluate the microwave assisted drug delivery of titanium dioxide/rose bengal chitosan based nanoparticles for micro-photo-dynamic skin cancer treatment both in vitro and in vivo.

## Materials and methods

### Materials

Every chemical utilized came from a commercial source and didn't require any additional purification. Titanium tetra isopropoxide (TTIP), Chitosan, tripolyphosphate (TPP), Acetic acid, N-ethyl-N′-(3-dimethyl aminopropyl) carbodiimide (EDC), sodium hydroxide (NaOH), and N-hydroxysuccinimide (NHS) were obtained from (Sigma-Aldrich). Rose Bengal were acquired from Shanghai, China's Aladdin Industries Inc. Kits of antioxidant total capacity (TAC), glutathione-S-transferase, peroxidase and reductase (GST, GPx, GSR), Superoxide dismutase (SOD), catalase (CAT), aspartate, alanine aminotransferase (AST, ALT), urea and creatinine, were acquired from Biodiagnostic Cairo, Egypt, along with lipid peroxide (Malondialdehyde; MDA). The ABT (spin column) total RNA Mini extraction kit, ABT cDNA synthesis H-minus kit, and WizPure™ qPCR (SYBR) Master were acquired from Wizbiosolutions Inc. and Applied Biotechnology, respectively.

**Fig. 1:**
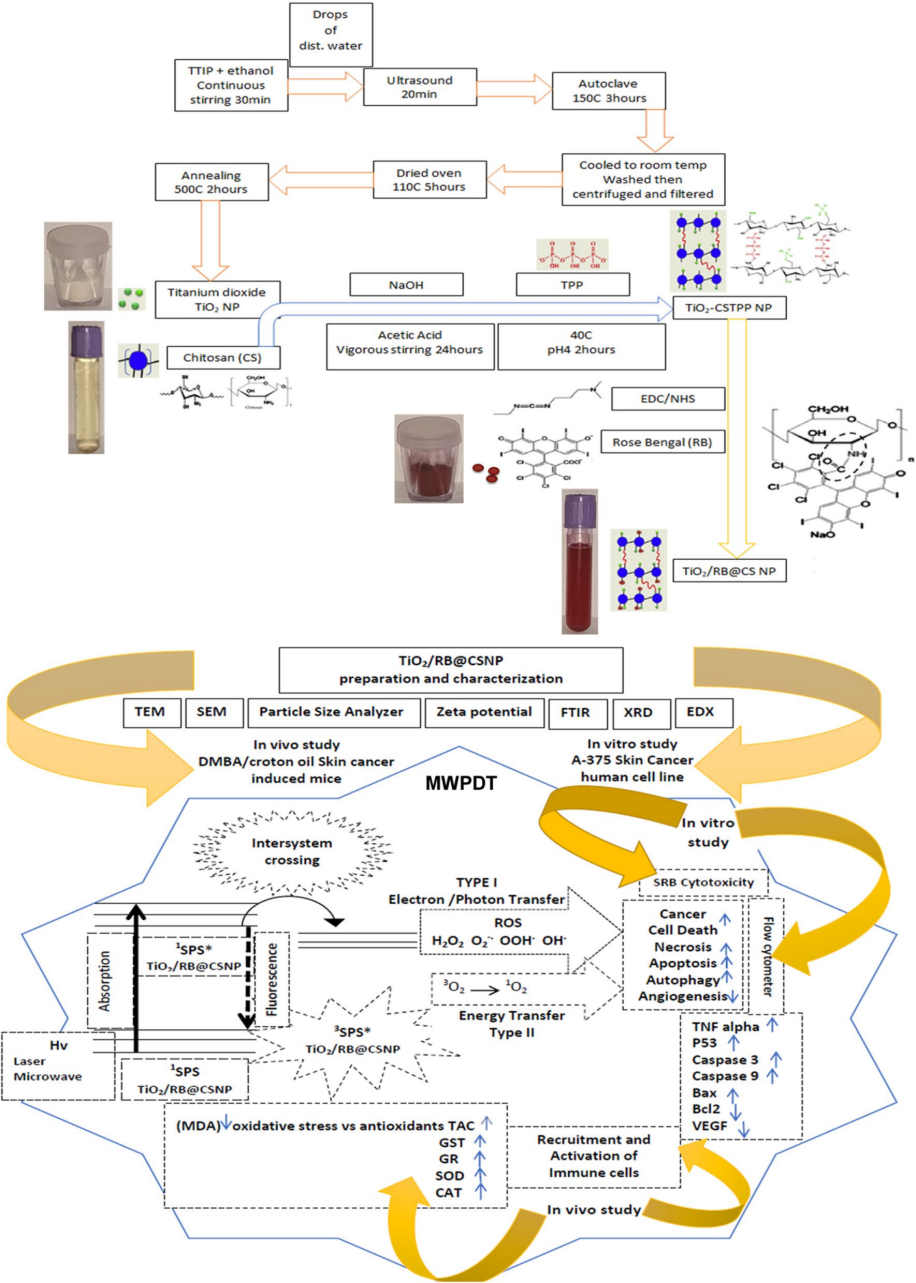
Schematic representation of TiO_2_/RB-CSNP synthesis and MWPDT

**Preparation and characterization of TiO**_**2**_/RB@CSNP: TiO_**2**_/RB-CSNP was utilized as MWPS in the current work Fig. (1). **TiO**_**2**_ **NP synthesis**; initially, 20 ml of ethanol solution are stirred continuously for 30 min to dissolve 0.1 N of TTIP. The dispersion medium was then created by adding a few drops of distilled water. The product spent twenty minutes in the ultrasonic bath. The solution was sonicated and then in an autoclave set at 150 °C placed for three hours. After the solution has reached room temperature, the contaminants were eliminated by centrifuging and washing it with deionized water. Filter paper is then used to filter it. The filtered sample was annealed for two hours at 500 °C after being dried for five hours at 110 °C. The resulting TiO_2_ nanoparticles were gathered and subjected to further analysis. **Chitosan NP synthesis;** 50 ml of 5% acetic acid solution was added with one gram of chitosan flakes. To guarantee the chitosan flakes full dissolution, the chitosan viscous solution was vigorously stirred for 24 h using a magnetic stirrer. The resulting viscous solution was poured into a 1000 mL (0.5 M) NaOH solution in drops at a rate of 1 mL/min using a 10 mL syringe. To get rid of any remaining traces of NaOH solution, distilled water was used to wash the fresh chitosan beads. **TiO**_**2**_**/RB@CSNP synthesis;** In order to initiate the crosslinking reaction, 90 mL of 1% TPP solution was added drop wise to the fresh chitosan beads while being heated in a water bath at 40 °C for two hours with moderate stirring. When cross-linking chitosan with TPP, the pH was adjusted to about 4. The cross-linked chitosan-tripolyphosphate beads (CSTPP) were then thoroughly cleaned with distilled water and allowed to air dry for a full day. Subsequently, the CSTPP beads were continually dried in the oven after being mashed with a mortar and pestle. The remaining CSTPP powder was intended for additional uses. Cross-linked chitosan-tripolyphosphate/TiO_2_ (TiO_2_-CSTPP) was created by loading TiO_2_ NP with chitosan flakes and then dissolving them in a 5% acetic acid solution in order to satisfy the optimization process's requirements. Conversely, RB was loaded onto chitosan after being chemically cross-linked to CSTPP using (5 mM) NHS and EDC. After dialyzing the TiO_2_/RB@CSNP for one week (using cellulose tubing from Sigma and cutting off 12,000–14000 g/mol), the filtrate was freeze-dried [16–18]. TiO_2_/RB-CSNP was characterized by scanning photoluminescence and absorbance spectrometry (PL, UV/Visible), Fourier transform infrared scanning spectroscopy (FTIR), scanning energy dispersive and diffraction X-ray (EDX,XRD), transmission and scanning electron microscopy (TEM, SEM), particle size analyzer and zeta potential to measure its size and shape. TiO_2_/RB@CSNP treated SKCA-A-375 cell lines and the DMBA/croton oil-SKCA-induced mice groups (painted topically) allowed to incubate for nine to twelve hours prior to being exposed to PDT and/or MWDT for two weeks.

## Methods

All applicable regulations and standards were adhered to during the entire investigation whole inquiry process.

### Ethics statement

Experimental procedures, animal handing and sampling followed the Guide for the Care and Use of Laboratory Animals and were approved by Research Ethical Committee [Institutional Animal Care and Use Committee, (ALEXU-IACUC; Code No.01219112821)] of the Medical Research Institute, Alexandria University (Alexandria, Egypt).

In vitro study: 1. (+ ve Control): SKCA cell line (A-375) was maintained in drug-free environment and were kept without treatment. 2. (TiO_2_/RB@CSNP without activation group): SKCA cell line (A-375) was treated with 0.033 µl TiO_2_/RB@CSNP only. 3. (Laser group): SKCA cell line (A-375) was exposed to laser, for 3 min. 4. (TiO_2_/RB@CSNP laser activated group): SKCA cell line (A-375) was treated with 0.033 µl TiO_2_/RB@CSNP and exposed to laser as group 3. 5. (Microwave group): SKCA cell line (A-375) was exposed to microwave for 3 min. 6. (TiO_2_/RB@CSNP microwave activated group): SKCA cell line (A-375) was treated with 0.033 µl TiO_2_/RB@CSNP and exposed to microwave as group 5. 7. (Laser and microwave combined group): SKCA cell line (A-375) was exposed laser and microwave for 3 min. 8. (TiO_2_/RB@CSNP laser and microwave combined activated group): SKCA cell line (A-375) was treated with 0.033 µl TiO_2_/RB@CSNP, exposed to laser and microwave as group 7.

In vivo study: Ninety Albino mice, with a weight range of 20 ± 5 g and of 60 ± 5 days, were acquired from the Faculty of Agriculture's animal house at Alexandria University. Mice were housed in appropriate cages with twelve wake-light/sleep-dark cycles and an ambient temperature of 25 ± 0.5 °C. The mice had unrestricted access to tap water and were fed a regular, consistent pellet diet. The medication was started after a week of acclimation for the mice. To put it briefly, nine groups of ten mice each were assembled from the mice: 1. (Negative Control group): Untreated normal, healthy mice were maintained. 2. (Positive SKCA Control group): mice were received topical painting of [(25 μg DMBA/0.1 ml acetone/mouse) twice weekly, then one week after the initiation with DMBA, (1% croton oil/0.1 ml acetone/mouse) was applied twice weekly] for eight weeks only to induce SKCA and maintained without treatment. 3. (TiO_2_/RB@CSNP without activation group): SKCA induced mice were treated by a daily dose of 0.033 ml of dissolved TiO_2_/RB@CSNP only. 4. (Laser exposed group): For two weeks, SKCA-induced mice were subjected to laser for three minutes every day. 5. (Laser activated TiO_2_/RB@CSNP group): SKCA induced mice were treated by a daily dose of 0.033 ml of TiO_2_/RB@CSNP per mouse and subsequently subjected to a laser for three minutes every day. 6. (Microwave exposed group): For two weeks, SKCA-induced mice were subjected to microwave for three minutes each day. 7. (Microwave activated TiO_2_/RB@CSNP group): SKCA-induced mice were treated by a daily dose of 0.033 ml of TiO_2_/RB@CSNP per mouse and subsequently subjected to microwave for three minutes every day. 8. (Laser and microwave combination activated group): For two weeks, SKCA-induced mice were subjected to laser light, followed by microwave for three minutes every day. 9. (Laser and microwave combined activated TiO_2_/RB@CSNP group): SKCA-induced mice were treated by a daily dose of 0.033 ml TiO_2_/RB@CSNP per mouse, and were exposed to both laser and microwave for three minutes each day.

### Instruments

#### Laser irradiation

A-375 cell line of each group was subjected to a three-minute laser therapy under the predetermined conditions in in vitro study section. For in vivo study; the mice were made anesthetic with 100, 10 mg/kg bw Ketamine and Xylazine respectively injection before to the laser exposure. The hair was shaved around the tumor. The mouse was placed with its back to the board. The probe was positioned almost atop the tumor exactly, and each group received a three-minute laser therapy under the predetermined conditions. After PDT, to prevent skin irritation mice were maintained in the dark. A laser LAS 250- Hi-Tech infrared diode type, fysiomed, China, with a peak 50 W output and a 600–904 nm wavelength of 7000 Hz frequencies, was utilized to expose the tumor in mice (10 mW and 0.43 J/cm2).

#### Microwave irradiation

A-375 cell line of each group was subjected to a three-minute microwave therapy under the predetermined conditions in in vitro study section. For in vivo study; the mice were put to sleep with 100, 10 mg/kg bw Ketamine and Xylazine respectively injection before to being exposed to microwave irradiation. The hair around the tumor was shaved. The mouse tumor was placed with its back to the board. The probe was positioned almost exactly atop the tumor, and each group was subjected to a three-minute microwave therapy under the predetermined conditions. Mice tumors were exposed using a microwave (Microwave Diathermy Model HME-009, China) operated at + 10 ~ + 70 ambient temperature, 30%−75%, relative humidity, 700 hPa ~ 1060 hPa atmospheric pressure, 220 ± 22 V 50 ± 1HZ power supply, 50 ± 1 Hz power frequency, rated output power: ≤ 100 W, output power ≤ 450 VA, 50 Ω matched load impedance).

When the study protocols was over, the mice were euthanized by inhaling 5% (overdose) isoflurane and immediately after 60 s as the animals no longer have a heartbeat and have whitish eyes, cervical dislocation was performed to ensure euthanasia. After the dissection, blood samples were taken in order to get whole blood and sera. One portion of the blood was centrifuged for ten minutes at 1000xg, and the separated sera were kept until analysis at −20 °C. The leftover blood sample was collected and maintained until the analysis of genes relative expressions identification was initiated at −80 °C. It was then transferred from the vial containing EDTA to another vial containing RNA later solution. Additionally, SKCA tissues were taken out right away, cleaned in cold saline, pierced in a vial with a needle, and kept for histological analysis in 10% formalin/saline.

#### Cell culture

Human skin cancer A-375 cell line was provided from the American Type Culture Collection (ATCC). The cells were cultured at 37 °C in a humidified 5% (v/v) CO_2_ environment in DMEM medium supplemented with 10% heat-inactivated fetal bovine serum, 100 units/mL of penicillin and100 mg/mL streptomycin.

#### Assay for cytotoxicity and cell viability

The cytotoxic effect of TiO_2_/RB@CSNP against A-375 cells was assessed using the sulforhodamine B (SRB) test. After 100 μL (5 × 10^3 cells) aliquots of cell suspension were planted on 96-well plates, the entire medium was incubated for 24 h. The cells were treated with a further aliquot of 100 μL media with TiO_2_/RB@CSNP at various doses (1000, 100, 10, 1.0, and 0.1 μM).After being exposed to various modalities for 24 h, with and without TiO_2_/RB@CSNP, the cells were fixed by replacing the media with 150 μL of 10% TCA and incubated for one hour at 4 °C. Following the removal of the TCA solution, the cells were washed five times with distilled water. Aliquots of 70 μL (0.4% w/v) SRB solution were added, and they were then left to sit at room temperature in a dark place for ten minutes. The plates were allowed to air dry for the full night following three washing with 1% acetic acid. The protein-bound SRB stain was then dissolved with 150 μL (10 mM) of TRIS, and the absorbance at 540 nm was measured using a LABTECH.®-FLUOstar BMG Omega microplate reader (Ortenberg, Germany) [19].

### Flow cytometer assay

#### Cell cycle analysis

After being treated with different modalities for 24 h, both with and without TiO_2_/RB@CSNP, A-375 cells (10^5^ cells) were collected by trypsinization. After that, the cells were twice cleaned in ice-cold PBS (pH 7.4). The cells were fixed for an hour at 4 °C after being re-suspended in two milliliters of 60% ice-cold ethanol. The fixed cells were first suspended in 1 mL of PBS with 10 µg/mL propidium iodide (PI) and 50 µg/mL RNAase A, and then they were repeatedly washed in PBS (pH 7.4). After incubating in the dark for 20 min at 37 °C, the cells were analyzed by flow cytometer using an ACEA Novocyte™ flow cytometer (ACEA Biosciences Inc., San Diego, CA, USA) equipped with a FL2 (λex/em 535/617 nm) signal detector to ascertain the DNA content of the cells. For every sample, a total of 12,000 events were collected. The cell cycle distribution was ascertained using ACEA NovoExpress.™ software (San Diego, ACEA Biosciences Inc., CA, USA) [20].

#### Analysis of necrosis and apoptosis in Annexin V-FITC/PI

The populations of necrosis and apoptosis cells were identified using two fluorescent channels flow cytometer in combination with the Abcam Inc., Cambridge Science Park, Cambridge, UK, Annexin V-FITC apoptosis detection kit. Following a 24-h period of treatment with various modalities both with and without TiO_2_/RB@CSNP, A-375 cells (10^5^ total) were isolated using trypsinization and twice washed in pH 7.4 ice-cold PBS. Cells were treated with 0.5 ml of Annexin V-FITC/PI solution for 30 min at room temperature in the dark, as per the manufacturer's instructions. Following staining, the fluorescence signals for FITC and PI were evaluated using FL1 and FL2 signal detectors (λex/em 488/530 nm for FITC and λex/em 535/617 nm for PI), respectively, using Novocyte™ ACEA flow cytometer. For each sample, a total of 12,000 events were recorded. Positive FITC and/or PI cells were computed and quantified using quadrant analysis using NovoExpress.™ ACEA software [21].

#### Autophagy analysis

Acridine orange (AO), a lysosomal dye, and flow cytometer analysis were used to quantify autophagic cell death. Trypsinization was used to extract A-375 cells (10^5^ cells) after different modalities were used for 24 h, both with and without TiO_2_/RB@CSNP. After that, the cells were twice cleaned in ice-cold PBS (pH 7.4). The cells were stained with acridine orange (10 µM) and then incubated at 37 °C in the dark for 30 min. After labeling, cells were injected using a Novocyte™ ACEA flow cytometer, and the acridine orange fluorescent signals were analyzed using the FL1 signal detector (λex/em 488/530 nm). For each sample, 12,000 events were gathered, and net fluorescence intensities (NFI) are measured using NovoExpress.™ ACEA software [22].

#### Migration (Wound healing) assay

Using a cell scratch assay, the effect of TiO_2_/RB@CSNP on A-375 cell migration was examined. A-375 cells were seeded onto a coated 12-well plate at a density of 2 × 10^5^/well for the scratch wound assay. After that, they were grown in 5% FBS-DMEM for the whole night at 37 °C and 5% CO_2_. The next day, horizontal scratches were applied to the confluent monolayer. After that, PBS was used to thoroughly clean the plate. New media containing activated TiO_2_/RB@CSNP was added to the treatment wells, and fresh medium was added to the control wells. At predetermined intervals, pictures were taken with an inverted microscope. The plate was incubated at 37 °C with 5% CO_2_ in between time intervals. Version 3.7 of the MII ImageView program displays and analyses the obtained images [23].

### Histological, molecular, and biochemical analyses

#### Examination of the Liver and Kidney Enzymes Biochemically

Using commercial kits, kidney (creatinine, and urea) and the liver (AST, and ALT) enzymes were tested in accordance with Burtis et al. [24].

#### Determination of the antioxidant markers and oxidative stress (oxidants)

The levels of antioxidant markers and oxidants in the serum were assessed using commercial kits. Malondialdehyde (MDA), which are implied to predicting lipid peroxidation [25]. SOD activity [26] glutathione- peroxidase, S-transferase, and reductase (GPx, GST, GR) activities [27, 28], Catalase activity [29, 30], Total antioxidant activity (TAC) [31].

#### Evaluation of Caspase (3, 9), p53, Bax, Bcl-2, TNF alpha, and VEGF relative gene expressions

QRT-PCR was used to determine genes expressions. Total RNA from blood samples was isolated using the ABT Total RNA Mini spin column extraction kit according to the directions. Purity by measuring the absorption 260/280 nm ratio was determined, which was usually greater than 1.8. As directed by the cDNA synthesis ABT H-minus kit, the cDNA was produced using an RT-PCR one-step reaction. For qRT-PCR, qPCR Master WizPure™ (SYBR) with ROX Dye was utilized. A reaction (20 µL) mixture using the template (5 µL) C-DNA and target gene primers (0.25 µM) P53-R: TGGAATCAACCCACAGCTGCA, CTGTCATCTTCTGTCCCTTC is P53-F, Caspase3-R: AAATGACCCCTTCATCACCA, TGTCATCTCGCTCTGGTACG is Caspase3-F, Caspase9-F: AGTTCCCGGGTGCTGTCTAT, GCCATGGTCTTTCTGCTCAC is Caspase9-R, TNF-α-R: TGAGATCCATGCCGTTGGC, CACGTCGTAGCAAACCACC is TNF-α-F: Bax-R: CCAGTTCATCTCCAATTCG, CTACAGGGTTTCATCCAG is Bax-F, Bcl2-F: GTGGATGACTGAGTACCT. CCAGGAGAAATCAAACAGAG is Bcl2-R, VEGF-R: TTTCTCCGCTCTGAACAAGG, AAAAACGAAAGCGCAAGAAA is VEGF-F, using qRT-PCR Sybr Green (10 µL) and β-actin-R (CTCTCAGCTGTGGTGGTGAA) and β-actin-F (AGCCATGTACGTAGCCATCC) was utilized. 35 cycles were employed in each run of PikoReal Thermo Scientific's qRT-PCR apparatus (PR0241401024), with 10 s at 95 °C, 10 s at 55 °C, and 5 s at 72 °C. The initial denaturation was done for five minutes at 95 °C. The identical sample β-actin mRNA levels were used to compare all of the gene expressions, and the fold difference was calculated using the Eq. 2^−ΔΔCt^ previously mentioned [32].

#### SKCA tissue histopathology analyses

After being embedded in paraffin, the gathered SKCA samples were sectioned into sections after being fixed by soaking them in a formalin/saline (10%) solution. To ascertain the extent of the histological changes in the SK tissue, eosin and hematoxylin (H&E) staining dye was used. Every slide was inspected and photographed using a light microscope [33].

**For statistical analysis**, the data was given as mean ± standard deviation (SD). One-way analysis of variance (ANOVA) was used to confirm the statistical variances of the data. To establish statistical significance, a *p* value of less than 0.05 was utilized. To compare groups, SPSS 25.0's post hoc analysis function was utilized.

## Results

### Characterization of TiO_2_/RB@CSNP

**Fig. 2:**
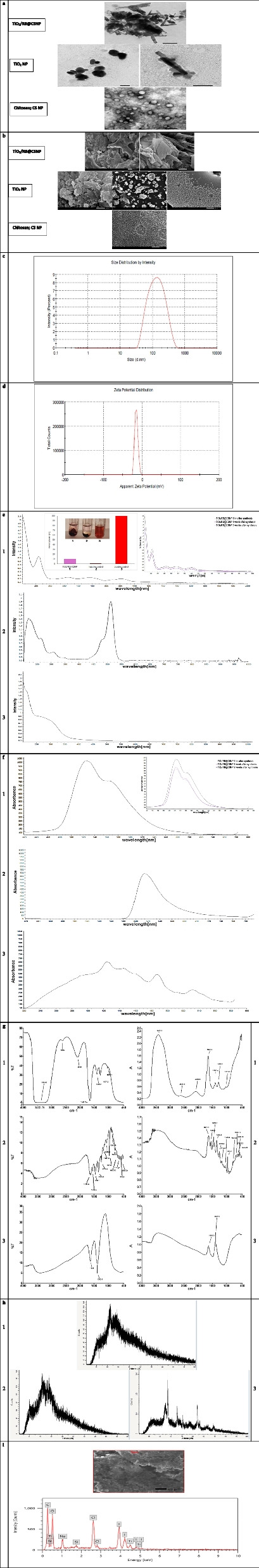
Characterization of TiO_2_/RB@CSNP; **a**. TEM, **b**. SEM, **c**. particle size, **d**. zeta potential of TiO_2_/RB@CSNP, **e**. UV–Vis spectra, **f**. PL, **g**. FTIR transmittance and absorbance, **h**. XRD of (1. TiO_2_/RB@CSNP, 2. RB, 3. TiO_2_), **i**. EDX of TiO_2_/RB@CSNP

In accordance with the particle size of TiO2/RB@CSNP demonstrated by TEM as well as particle size analyzer Fig. (2a,c), SEM results showing porous surface morphology of TiO_2_, CSNP and illustrating the conjugation of TiO_2_/RB to CSNP Fig. (2b), zeta potential results Fig. (2d), optimum characteristic (UV–Visible) absorption peaks for TiO_2_ and RB before and after conjugation with CSNP Fig. (2e), optimum characteristic (PL) photoluminescence peaks for TiO_2_ and RB before and after conjugation with CSNP Fig. (2f), characteristic FTIR bands found that match the vibrations of the functional groups that are present in TiO_2_ and RB before and after conjugation with CSNP represented on Fig. (2g); the FTIR spectra of TiO_2_ in the range of 450–4000 cm^−1^, the peaks at 3432.8 and 1638 cm^−1^ in the spectra are due to the stretching and bending vibration of the -OH group. In the spectrum of pure TiO_2_, the peaks at 523.88 cm^−1^ show stretching vibration of Ti–O and peaks at 1382 cm^−1^ shows stretching vibrations of Ti–O-Ti. RB-FTIR peaks at (1631.8, 1556.1, 1452.1, 1343.4, 1239.4, 1163.8, 1045.5, 955.8, 761.98, 670.72, 662.72, 568.18, 535.09) peak near 1614 cm^−1^ could be attributed to carbonyl stretching (C–O) vibrations and the other three strong peaks in the ranges of 1300–1600 cm^−1^ correspond to the (C–C) stretching vibrations. The pure CS FTIR spectrum several characteristic peaks at 3360, 2919, 2874, 1641.2, 1592, 1395.6, 127.2, 1153, 1017.2 and 893 cm^−1^, around 901 and 1155 cm^−1^, assigned to the saccharine structure and an amino and amid characteristic peak at 1592 cm^−1^. There was a stronger absorption band at 1641 corresponding to the carbonyl of CS, as well as characteristic XRD peaks observed at 2θ value of TiO_2_ 2θ at peak (25.46^°^, 27^°^, 36.32^°^, 40.79^°^, 44.89^°^, 54.91^°^, 55.75^°^, 67.63^°^, 68.38^°^) and RB 2θ at peak (7.82^°^, 8.48^°^, 12.25^°^, 14.68^°^, 16.25^°^, 18.267^°^, 20.61^°^, 22.59^°^, 26.85.^°^) before and after conjugation with CSNP exhibit very broad peaks at 2θ = 10° and 2θ = 20° indicating crystalnity and phase purity of prepared TiO_2_/RB@CSNP CSNP Fig. (2 h), and EDX elemental analysis illustrating the presence of C in the CSNP in correct state alongside with Ti, O, N TiO_2_/RB content Fig. (2i), demonstrating the correct and well preparation of TiO_2_/RB@CSNP as well as correct conjugation and nanocomposite formation in nanoscale. The overall results indicated that consistent with earlier research, TiO_2_/RB@CSNP was synthesized successfully [16–18].

### Cytotoxicity of PDT, MWDT and MWPDT in presence and absence of (TiO_2_/RB@CSNP) upon treatment of SKCA A-375 cell line

**Fig. 3:**
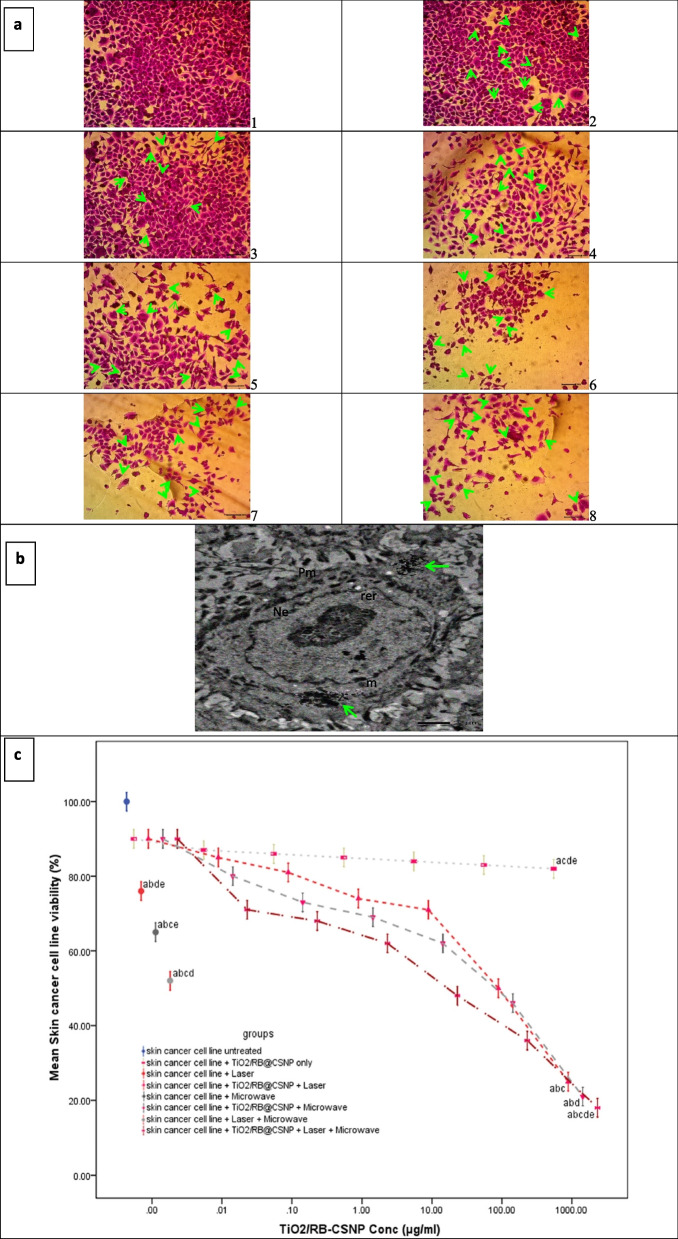
The effect of different treatment modalities on skin cancer (A-375) cell viability; Cells were exposed to different treatment modalities with serial dilution of TiO_2_/RB@CSNP for 24 h, **a.** microscopic investigations, **b.** A375 containing TiO_2_/RB@CSNP (arrow), Ne; nuclear envelop, Pm; plasma membrane, m; mitochondria, rer; rough endoplasmic reticulum and **c.** dose response curve in all in vitro study groups; cell viability was determined using SRB assay. cell viability (%): F = 5.822 *p* < 0.001*. Mean ± SD is used to illustrate the results (*n* = 3). F stands for the ANOVA test value. ^a,b,c,d,e^ Significance to (skin cancer untreated group, TiO_2_/RB@CSNP treated non activated group, laser-, microwave-, laser + microwave- exposed groups)

The cytotoxic effect of different activation modalities with and without TiO_2_/RB@CSNP was investigated in SKCA A-375 cells after 24 h treatments using the SRB viability assay. The SKCA A-375 cell line underwent treatment with varying TiO_2_/RB@CSNP doses and different activation modalities, which led to a rise in the number of floating cells and a change in cellular morphology. Additionally, the SRB test was used to evaluate PDT, MWDT, and MWPDT in the presence and absence of TiO_2_/RB@CSNP cytotoxicity, our findings revealed that the TiO_2_/RB@CSNP decrease proliferation of SKCA A-375 cell in a dose-dependent manner. In addition, the cytotoxicity analysis results of the current study manifested that the SKCA A-375 cell line was slightly affected by treatment with TiO_2_/RB@CSNP without activation followed by the SKCA A-375 cell line treated with lasers, microwave without TiO_2_/RB@CSNP. The SKCA A-375 cells cytotoxic effectiveness and growth inhibition owing to laser (PDT) and the microwave (MWDT) are both improved by the presence of TiO_2_/RB@CSNP. The acquired results demonstrated that both with and without the TiO_2_/RB@CSNP, MW is more cytotoxic effective than IRL against SKCA A-375 cell line. For integration with IRL, MW was chosen. In comparison to employing IRL or MW alone, the combination therapy approach (MWPDT) in presence of TiO_2_/RB@CSNP is most cytotoxic effective against SKCA A-375 cell line. Plotting cell viability vs. concentration yielded the concentration at which the viable cells inhibited 50% by TiO_2_/RB@CSNP (IC50), which was determined in μg/ml Fig. (3).

### Cell cycle distribution effect of PDT, MWDT and MWPDT in presence and absence of (TiO_2_/RB@CSNP) upon treatment of SKCA A-375

**Fig. 4:**
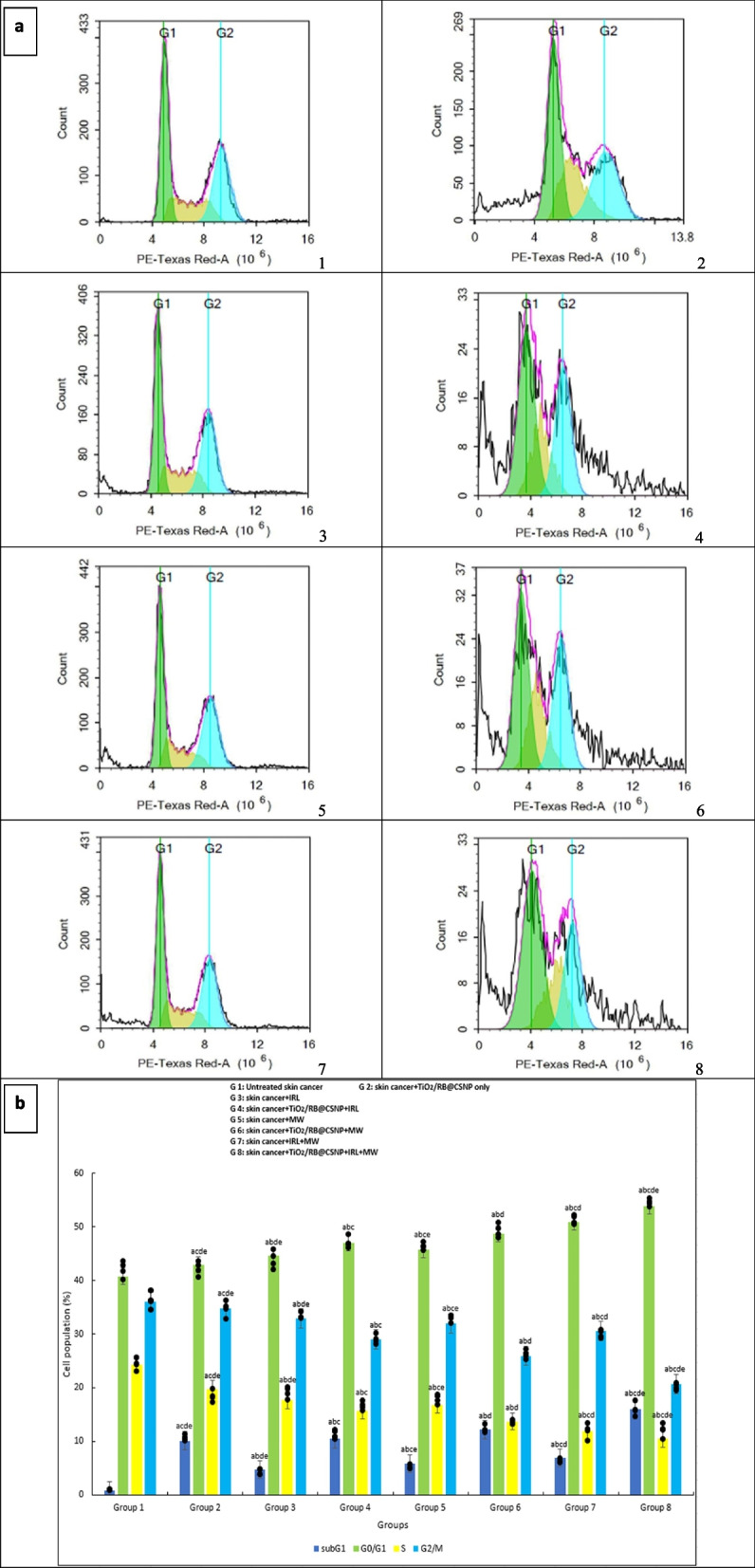
The effect of different treatment modalities on skin cancer (A-375) cell cycle distribution in all in vitro study groups; 1. Cells were exposed to different treatment modalities for 24 h, 2. Cell cycle distribution was determined using DNA cytometry analysis and different cell phases were plotted as percentage of total events; SubG1(%): F = 329.212 *p* < 0.001*, G0/G1(%): F = 88.084 *p* < 0.001*, S(%): F = 92.935 *p* < 0.001*, G2/M(%): F = 173.137 *p* < 0.001*. Mean ± SD is used to illustrate the results (*n* = 3). F stands for the ANOVA test value..^a,b,c,d,e^ Significance to (skin cancer untreated group, TiO_2_/RB@CSNP treated non activated group, laser-, microwave-, laser + microwave- exposed groups)

When SKCA A-375 cells are treated with TiO_2_/RB@CSNP at an IC50 equivalent concentration, a rise in the cell population at the G1 phase has been observed. The SKCA A-375 cell line was slightly affected by treatment with TiO_2_/RB@CSNP without activation exhibited increase the cell population at the G1 phase and resulted in cell death manifested by Sub G1 phase increase and reciprocally, caused S phase and G2/M phase decrease followed by the SKCA A-375 cell line treated with lasers, microwave without TiO_2_/RB@CSNP. The effectiveness SKCA A-375 cells distribution, arrest and growth inhibition owing to laser (PDT) and the microwave (MWDT) are both improved by the presence of TiO_2_/RB@CSNP exhibited increase the cell population at the G1 phase and resulted in cell death manifested by Sub G1 phase increase and reciprocally, caused S phase and G2/M phase decrease. The acquired results demonstrated that both with and without the TiO_2_/RB@CSNP, RF is more effective than IRL against SKCA A-375 cell line. For integration with IRL, MW was chosen. In comparison to employing IRL or MW alone, the combination therapy approach (MWPDT) in presence of TiO_2_/RB@CSNP is most effective against SKCA A-375 cell line exhibited cell population increase at the G1 phase and resulted in cell death manifested by Sub G1 phase increase and reciprocally, caused S phase and G2/M phase decrease Fig. (4).

### Apoptosis and necrosis cell death mechanism of PDT, MWDT and MWPDT in presence and absence of (TiO_2_/RB@CSNP) upon treatment of SKCA A-375

**Fig. 5:**
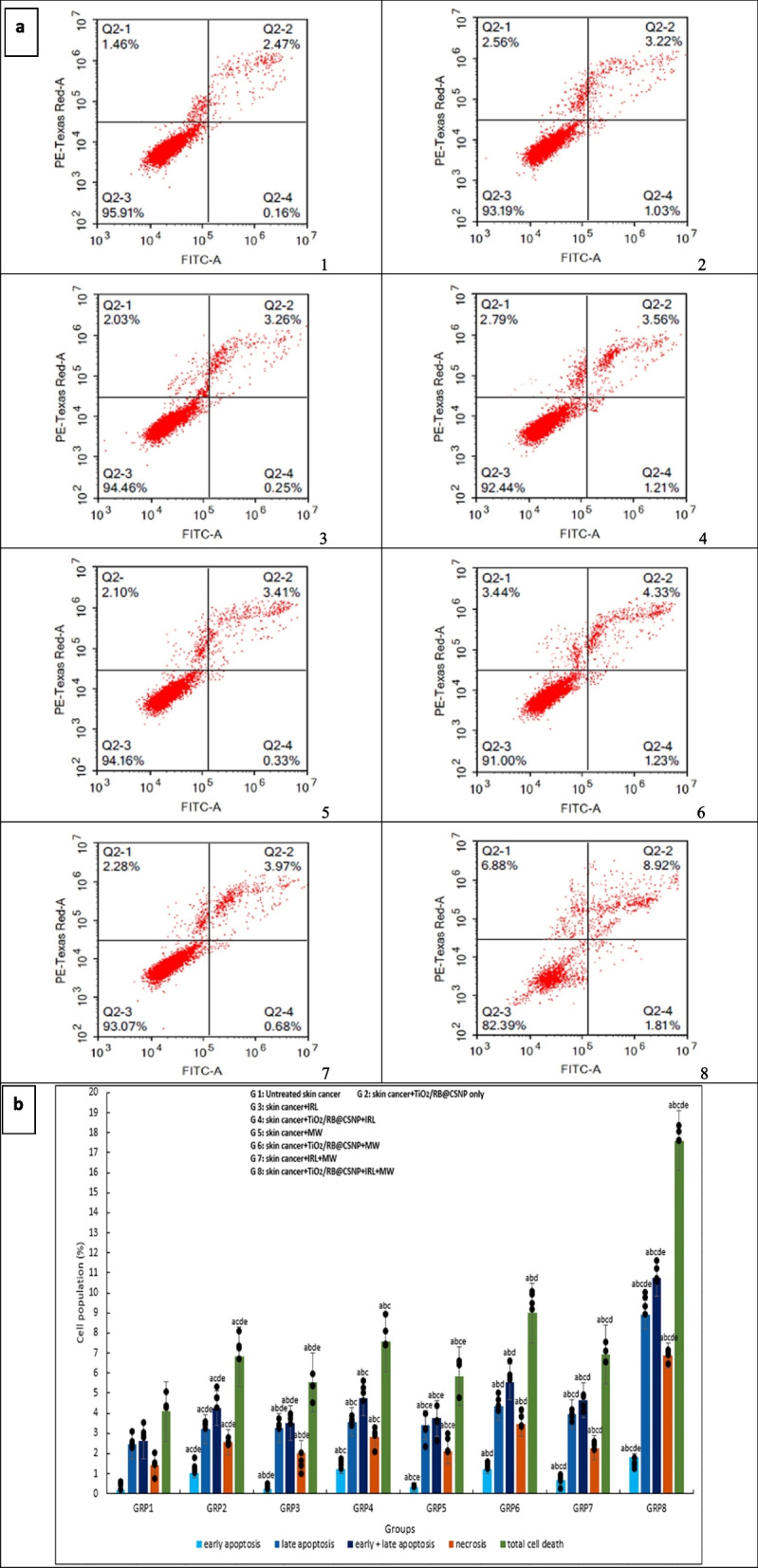
The effect of different treatment modalities on skin cancer (A-375) apoptosis and necrosis in all in vitro study groups; 1. Cells were exposed to different treatment modalities for 24 h, 2. Cells were stained with annexin V-FITC/PI and different cell populations were plotted as percentage of total events. early apoptosis (%): F = 16.884 *p* < 0.001*, late apoptosis (%): F = 26.304 *p* < 0.001*, early and late apoptosis (%): F = 23.439 *p* < 0.001*, necrosis (%): F = 18.719 *p* < 0.001*, total cell death (%): F = 23.288 *p* < 0.001*. Mean ± SD is used to illustrate the results (*n* = 3). F stands for the ANOVA test value..^a,b,c,d,e^ Significance to (skin cancer untreated group, TiO_2_/RB@CSNP treated non activated group, laser-, microwave-, laser + microwave- exposed groups)

The SRB test and microscopic examination point to an apoptotic reaction to TiO_2_/RB@CSNP as the possible cause of the decrease in cell viability. To gain additional knowledge about the mechanism underlying cell death (apoptosis versus necrosis), SKCA A-375 cells were treated with different activation modalities in presence and absence of TiO_2_/RB@CSNP for 24 h. The treated cells'flow cytometer histogram revealed a population shift that progressed from living cells in the lower left quadrant to early apoptosis in the lower right and finally from late apoptosis in the upper right quadrant to cell necrosis in the upper left. The treated cells also showed increase of annexin V positivity. SKCA A-375 cells treated with either TiO_2_/RB@CSNP without activation or with lasers and microwave without TiO_2_/RB@CSNP showed a substantial elevation in the percentage of cells exhibiting early and late apoptosis. In SKCA A-375 cells treated with laser (PDT) and microwave (MWDT), the addition of TiO_2_/RB@CSNP increases both the elevation in the percentage of cells with early and late apoptosis. The obtained data showed that MW is more effective than IRL at raising the proportion of SKCA A-375 cells with early and late apoptosis, both with and without the TiO_2_/RB@CSNP. For integration with IRL, MW was chosen. The combination MWPDT therapy approach in TiO_2_/RB@CSNP presence is the most effective way to increase the proportion of SKCA A-375 cells with early and late apoptosis, as compared to using IRL or MW alone. Comparable observations were found for the percentage of necrotic cells in SKCA A-375 cells treated with either TiO_2_/RB@CSNP without activation or with lasers and microwave without TiO_2_/RB@CSNP. The inclusion of TiO_2_/RB@CSNP improves both the elevation in the necrotic cells percentage of SKCA A-375 cells treated with microwave (MWDT) and laser (PDT). The obtained data showed that MW is more effective than IRL at increasing the proportion of necrotic SKCA A-375 cells, both with and without the TiO_2_/RB@CSNP. The combined therapy strategy (MWPDT) in the presence of TiO_2_/RB@CSNP is most successful in elevating the percentage of SKCA A-375 cells with necrosis as compared to using IRL or MW alone Fig. (5).

### Autophagy cell death mechanism of PDT, MWDT and MWPDT in presence and absence of (TiO_2_/RB@CSNP) upon treatment of SKCA A-375

**Fig. 6:**
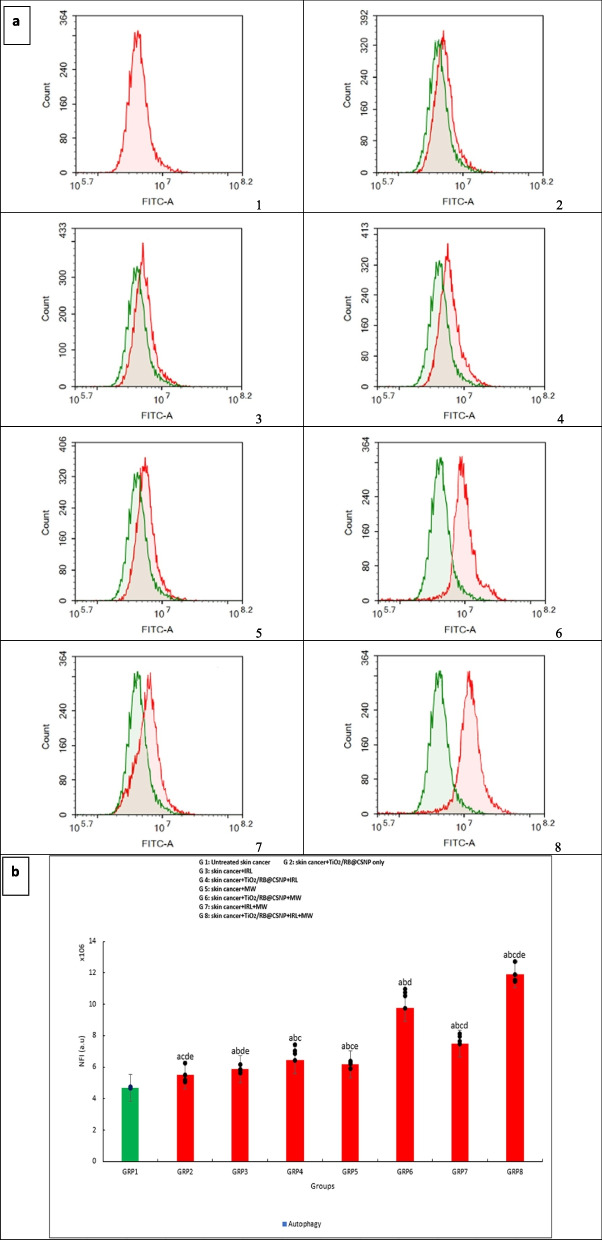
The effect of different treatment modalities on skin cancer (A-375) autophagy in all in vitro study groups; 1. Cells were exposed to different treatment modalities for 24 h; and were stained with Cyto-ID autophagosome tracker. 2. Net fluorescent intensity (NFI; red color) were plotted and compared to basal fluorescence of control group (green color). autophagy (%): F = 24.876 *p* < 0.001*. Mean ± SD is used to illustrate the results (*n* = 3). F stands for the ANOVA test value..^a,b,c,d,e^ Significance to (skin cancer untreated group, TiO_2_/RB@CSNP treated non activated group, laser-, microwave-, laser + microwave- exposed groups)

The SRB assay and microscopic examination indicate that program cell death other than apoptosis by autophagy in response to TiO_2_/RB@CSNP may be the cause cell viability decline. For further exploration the process of cell death (autophagy), SKCA A-375 cells were subjected to different activation modalities for duration of 24 h, both in the presence and absence of TiO_2_/RB@CSNP. The treated cells expressed redder and less green when AO, a fluorophore that accumulates at high concentrations in acidic vesicular organelles (AVO) such as auto-lysosomes, dimerizes and causes a green to red metachromatic shift, that could be measured to study autophagy. SKCA A-375 cells treated with either TiO_2_/RB@CSNP without activation or with lasers and microwave without TiO_2_/RB@CSNP showed a substantial elevation in the percentage of cells undergoing autophagy. The presence of TiO_2_/RB@CSNP increases the percentage of autophagous cells in SKCA A-375 cells treated with both microwave dynamic therapy (MWDT) and laser photodynamic therapy (PDT). The obtained data showed that MW is more effective than IRL at raising the percentage of SKCA A-375 cells undergoing autophagy, both with and without the TiO_2_/RB@CSNP. For integration with IRL, MW was chosen. The combined therapy strategy (MWPDT) in the presence of TiO_2_/RB@CSNP is most successful in elevating the percentage of SKCA A-375 cells undergoing autophagy as compared to using IRL or MW alone Fig. (6).

### Cell migration inhibition mechanism of PDT, MWDT and MWPDT in presence and absence of (TiO_2_/RB@CSNP) upon treatment of CRCA SW-620

**Fig. 7:**
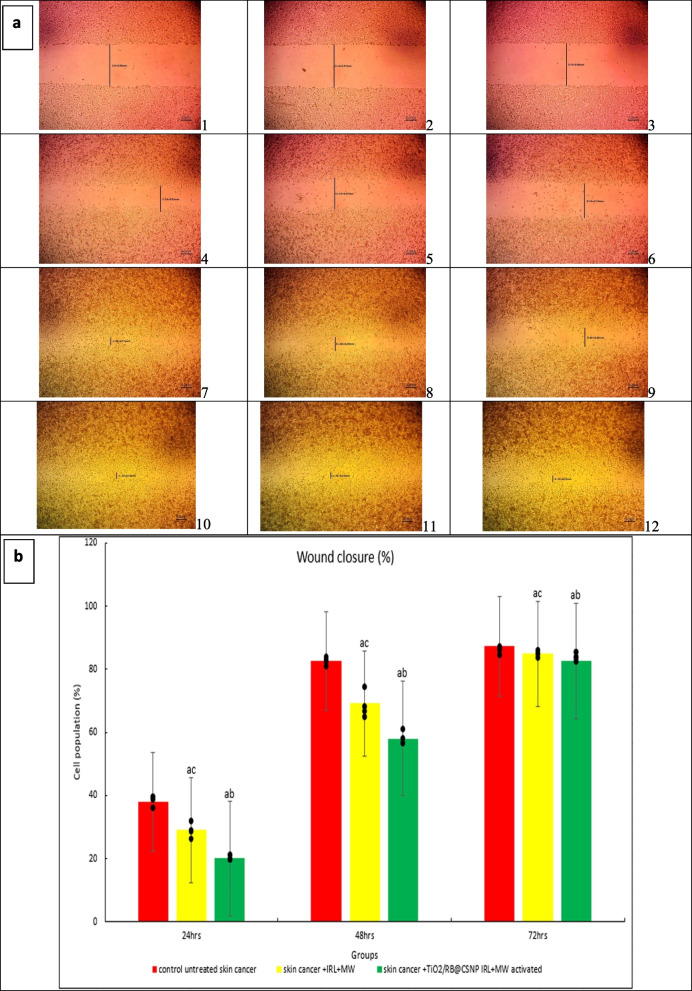
The effect of different treatment modalities on skin cancer (A-375) migration; 1. A-375 untreated, 2. A-375 treated with laser + microwave only, 3. A-375 treated with TiO_2_/RB@CSNP + laser + microwave. wound clousure-24 h (%): F = 2.416E3 *p* < 0.001*, wound clousure-48 h (%): F = 694.712 *p* < 0.001*, wound clousure-72 h (%): F = 2.855E3 *p* < 0.001*. Mean ± SD is used to illustrate the results (*n* = 3). F stands for the ANOVA test value..^a,b,c^ Significance to (skin cancer untreated group, laser + microwave only group, laser + microwave + TiO_2_/RB@CSNP groups)

After 24 h of treatment, SKCA A-375 cells were used to study the inhibitory effect of various activation methods with and without TiO_2_/RB@CSNP on cell migration. Daily wound closure assessments were conducted using the scratch assay until the control, untreated cells closed after 72 h. Compared to the untreated control group, the SKCA A-375 cell line treated with the combination therapy strategy (MWPDT) in the presence of TiO_2_/RB@CSNP shown a significant decrease in SKCA A-375 cell migration, illustrating a possible anti-migratory impact Fig. (7).

### (TiO_2_/RB@CSNP) of PDT, MWDT, MWPDT and oxidative stress in vivo SKCA models

**Fig. 8:**
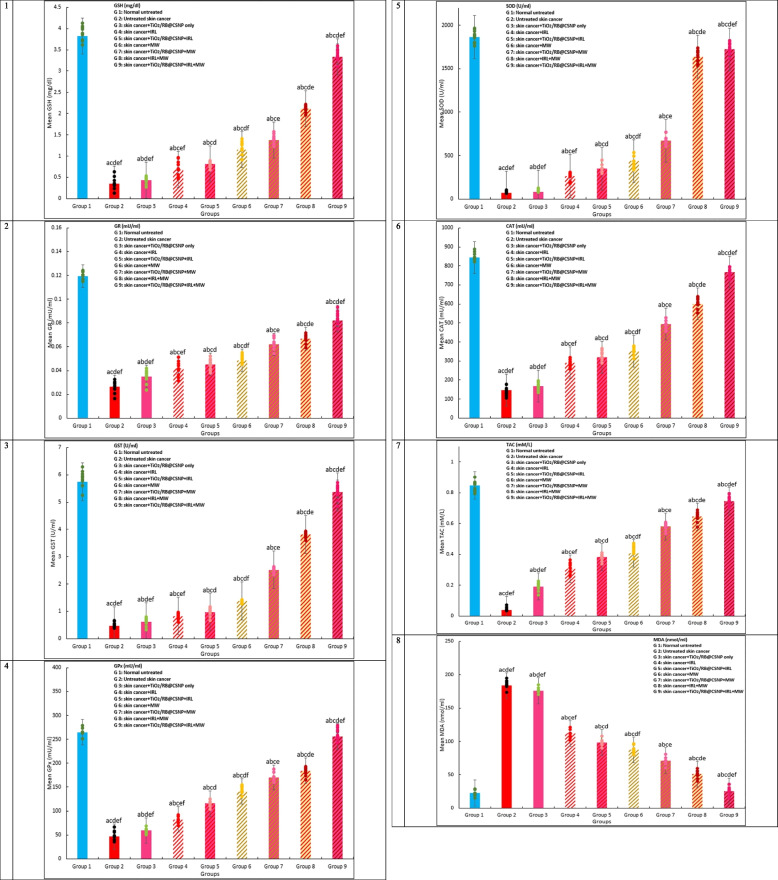
The effect of different treatment modalities on MDA, antioxidants activities, and capacities in all study groups; F: value for ANOVA test 1. GSH(mg/dl): F = 871.530 *p* < 0.001*, 2. GR (mU/ml): F = 161.065 *p* < 0.001*, 3. GST (U/ml): F = 1.221E3 *p* < 0.001*, 4. GPx (mU/ml): F = 4.155E4 *p* < 0.001*, 5. SOD (U/ml): F = 3.819E6 *p* < 0.001*, 6. CAT(mU/ml): F = 1.676E5 *p* < 0.001*, 7. TAC(mM/L): F = 453.839 *p* < 0.001*, 8. MDA(nmol/ml): F = 9.736E4 *p* < 0.001*. Mean ± SD is used to illustrate the results (*n* = 10). F stands for the ANOVA test value..^a,b,c,d,e^ Significance to (skin cancer untreated group, TiO_2_/RB@CSNP treated non activated group, laser-, microwave-, laser + microwave- exposed groups)

Figure (8) displays the effects of TiO_2_/RB@CSNP; PDT, MWDT, and MWPDT on lipid peroxidation oxidative stress MDA parameter in each of the experimental groups'mice. When comparing the untreated DMBA/croton oil-SKCA-induced control mouse group to the non-irradiated group of mice treated with TiO_2_/RB@CSNP alone, the MDA showed very modest alterations in the sera. The untreated DMBA/croton oil-SKCA-induced control animals had significantly greater values of this parameter compared to the control normal healthy mice. Furthermore, all DMBA/croton oil-SKCA-induced mice treated with laser, microwave, or laser and microwave only combination groups exhibited a substantial rise in concentrations of MDA relative to the control normal mice. In contrast to the DMBA/croton oil-SKCA-induced control animals, MDA levels were much lower in the IRL, MW, and combination of IRL and MW activated groups when TiO_2_/RB@CSNP was given, but this effect did not reach the level of control normal mice.

### (TiO_2_/RB@CSNP) PDT, MWDT, MWPDT and the antioxidant system in vivo SKCA models

Figure 8 illustrates how TiO_2_/RB@CSNP, PDT, MWDT, and MWPDT affected the antioxidant indicators (Catalase, SOD, TAC, GR, GPx and GST) in each of the research groups of mice. The DMBA/croton oil-SKCA-induced control group was compared to the TiO_2_/RB@CSNP alone without activation. Only marginally significant alterations were seen in the TAC level, GPx, GST, GR, SOD, and catalase activities. The untreated control DMBA/croton oil-SKCA-induced mice exhibited considerably lower levels of GPx, GST, GR, SOD, Catalase, and TAC than the normal control mice. Furthermore, regarding to the healthy control group of mice, all DMBA/croton oil-SKCA-induced mouse groups treated with laser, microwave, or laser and microwave combination solely exhibited a significant reduction in TAC levels as well as GPx, GST, GR, SOD, and Catalase activities. Activated TiO_2_/RB@CSNP in the IRL, MW, and combination of IRL and MW groups demonstrated a considerable elevation in the TAC level and the activities of Catalase, SOD, GR, GPx, and GST while did not approach the level of normal control group levels, in contrast to the untreated DMBA/croton oil-SKCA-induced control mice.

### (TiO_2_/RB@CSNP) PDT, MWDT, MWPDT treatment improved liver functions in vivo SKCA models

**Fig. 9:**
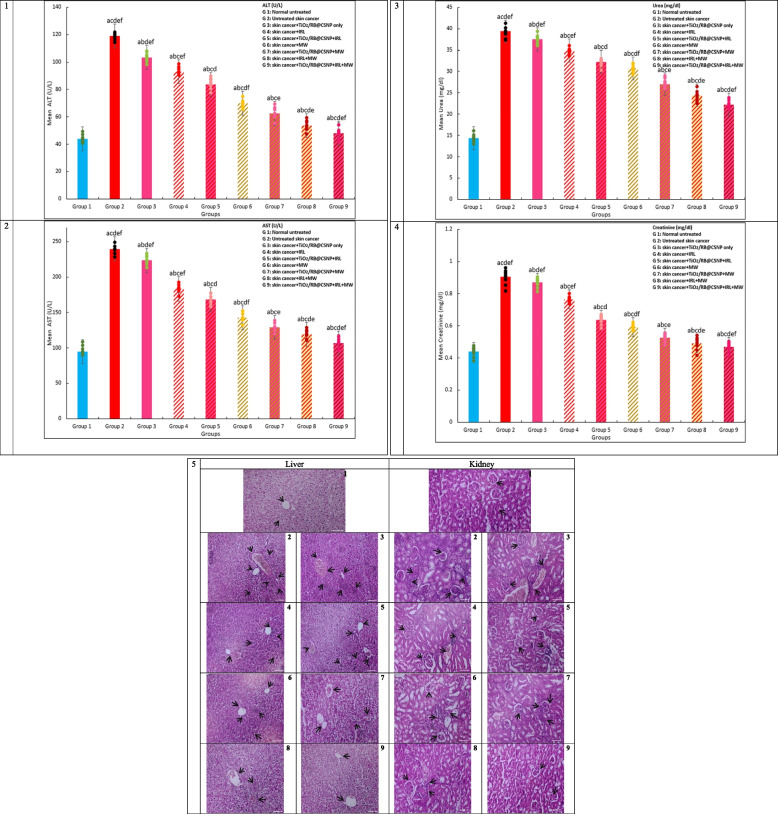
The effect of different treatment modalities on hepatic, renal biomarkers biomarkers and tissues histopathology in all study groups; F: value for ANOVA test 1. ALT (U/l): F = 7.865E3 *p* < 0.001*, 2. AST (U/l): F = 1.651E5 *p* < 0.001*, 3. Urea (mg/dl): F = 4.230E3 *p* < 0.001*, 4. Creatinine (mg/dl): F = 401.771 *p* < 0.001*. 5. H&E stained liver and kidney tissues; (1. Normal untreated group, 2. DMBA/croton oil induced SKCA group without any treatment, 3. DMBA/croton oil induced SKCA group treated with TiO_2_/RB@CSNP without activation, 4. DMBA/croton oil SKCA induced group subjected to laser only, 5. DMBA/croton oil SKCA induced group subjected to laser in TiO_2_/RB@CSNP presence, 6. DMBA/croton oil induced SKCA group subjected to microwave only, 7. DMBA/croton oil SKCA induced group subjected to microwave in TiO_2_/RB@CSNP presence, 8. DMBA/croton oil SKCA induced group subjected to combined modalities laser/microwave only, 9. DMBA/croton oil SKCA induced group subjected to combined modalities laser/microwave in TiO_2_/RB@CSNP presence. Mean ± SD is used to illustrate the results (*n* = 10). F stands for the ANOVA test value..^a,b,c,d,e^ Significance to (skin cancer untreated group, TiO_2_/RB@CSNP treated non activated group, laser-, microwave-, laser + microwave- exposed groups)

Figure (9) shows the results of the function liver tests performed on each group under study. While the sera levels of the untreated DMBA/croton oil-SKCA-induced control group were significantly higher than those in the normal control healthy mice, the levels of AST and ALT in the mice treated with TiO_2_/RB@CSNP alone without irradiation showed only marginally different changes from the untreated DMBA/croton oil-SKCA-induced control group. Furthermore, all DMBA/croton oil-SKCA-induced mice treated with laser, microwave, or of laser and microwave combination only groups displayed a significantly higher of AST and ALT level in comparison to the control normal group. Furthermore, as comparison to the untreated DMBA/croton oil-SKCA-induced control mice, the level of AST and ALT was dramatically reduced when TiO_2_/RB@CSNP was given to the IRL, MW, and combination of IRL and MW activated groups; nevertheless, the normal control group levels was not reached.

### (TiO_2_/RB@CSNP) PDT, MWDT, MWPDT treatment improved kidney functions in vivo SKCA models

Figure (9) shows the results of the function kidney tests performed all the groups under study. When compared to the untreated control DMBA/croton oil-SKCA-induced mice, the serum levels of creatinine or urea in mice treated with TiO_2_/RB@CSNP alone without irradiation showed only marginally significant changes; however, this parameter levels in the untreated control DMBA/croton oil-SKCA-induced mice were higher significantly when compared to the control healthy normal mice. Furthermore, all DMBA/croton oil-SKCA-induced mice treated with laser, microwave, or laser and microwave combination only groups showed a statistically significant elevation in creatinine and urea levels relative to the control normal group. Furthermore, when compared to the untreated control DMBA/croton oil-SKCA-induced mice, the administration of TiO_2_/RB@CSNP in the IRL, MW, and combination of IRL and MW activated groups considerably reduced the levels of creatinine and urea; however, the normal control group levels was not reached.

### (TiO_2_/RB@CSNP) PDT, MWDT, MWPDT anticancer, antiproliferative and antiangiogenic effects in vivo SKCA models

**Fig. 10:**
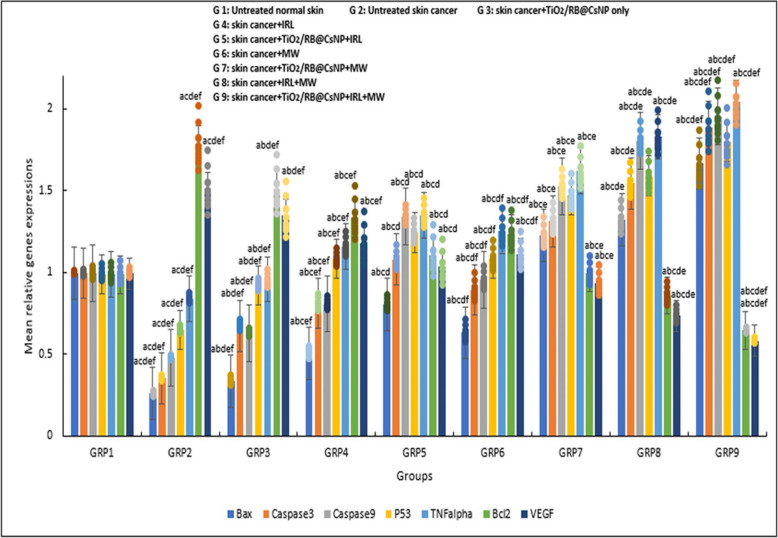
The effect of different treatment modalities on VEGF, TNF alpha, Bax, Caspase (9,3), Bcl-2 and p53, qRT-PCR relative genes expressions in all study groups; F: value for ANOVA test. p53: F = 406.566 *p* < 0.001*, Bax: F = 985.443 *p* < 0.001*, Caspase 9: F = 1.053E3 *p* < 0.001*, Caspase 3: F = 761.080 *p* < 0.001*, TNFalpha: F = 435.687 *p* < 0.001*, VEGF: F = 147.444 *p* < 0.001*, Bcl-2: F = 191.970 *p* < 0.001*. Mean ± SD is used to illustrate the results (*n* = 10). F stands for the ANOVA test value..^a,b,c,d,e^ Significance to (skin cancer untreated group, TiO_2_/RB@CSNP treated non activated group, laser-, microwave-, laser + microwave- exposed groups)

Figure 10 shows the effects of TiO_2_/RB@CSNP on the genes relative expressions of VEGF, TNF alpha, Bax, Caspase (3, 9), Bcl-2, and p53 in all groups under study. When TiO_2_/RB@CSNP was administered to mice in isolation without activation, the expression of VEGF, TNF alpha, Bax, Caspase (3, 9), Bcl-2, and p53 changed only slightly in comparison to the untreated control DMBA/croton oil-SKCA-induced group. In contrast, the untreated control DMBA/croton oil-SKCA-induced group's levels of caspase (3, 9), TNF alpha, Bax, and p53 were declined significantly, while those of Bcl-2, and VEGF were elevated significantly than those of control normal, healthy mice. Furthermore, regarding to the healthy control group of mice, all DMBA/croton oil-SKCA-induced mice treated with laser, microwave, or laser and microwave combination only groups exhibited significantly elevated levels of VEGF and Bcl-2 gene expressions and significantly decreased levels of TNF alpha, caspase (3, 9), Bax, and p53 gene expressions. The delivery of TiO_2_/RB@CSNP to the IRL, MW, and combination of IRL and MW activated groups resulted in significant increases in the TNF alpha, caspase (3, 9), Bax, and p53 genes expressions, and significant decreases in the expressions of the genes VEGF and Bcl-2, as compared to the control untreated DMBA/croton oil-SKCA-induced group.

### (TiO_2_/RB@CSNP) PDT, MWDT, MWPDT histopathological effect on in vivo SKCA models

**Fig. 11:**
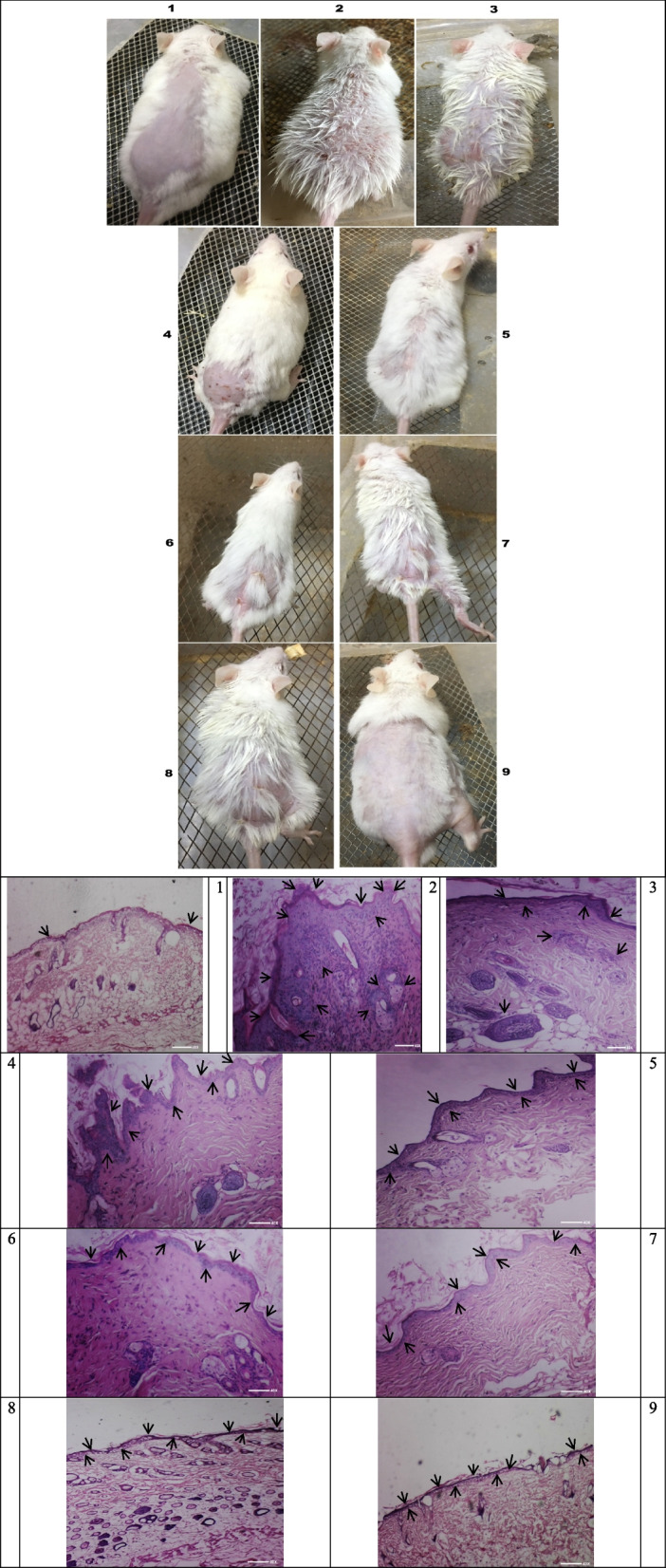
The in vivo study groups images and H&E skin tissue stained section demonstrating the effect of different treatment modalities on cellular level in all study groups; 1. Normal untreated group, 2. DMBA/croton oil induced SKCA group without any treatment, 3. TiO_2_/RB@CSNP treated group without activation, 4. DMBA/croton oil SKCA induced group subjected to laser only, 5. DMBA/croton oil SKCA induced group subjected to laser in presence of TiO_2_/RB@CSNP, 6. DMBA/croton oil induced SKCA group subjected to microwave only, 7. DMBA/croton oil SKCA induced group subjected to microwave in presence of TiO_2_/RB@CSNP, 8. DMBA/croton oil SKCA induced group subjected to combined modalities laser/microwave only, 9. DMBA/croton oil SKCA induced group subjected to combined modalities laser/microwave in presence of TiO_2_/RB@CSNP

Figure (11) displays the H&E stained tissue sections from all study mouse groups that demonstrate how TiO_2_/RB@CSNP, PDT, MWDT, and MWPDT affect SKCA caused by DMBA/croton oil. According to the histological study, all of the tumors in the untreated control DMBA/croton oil-SKCA-induced group exhibited 5% necrosis and were completely composed of extremely malignant cells. In comparison to the untreated control DMBA/croton oil-SKCA-induced mice, the histologically induced SKCA tissues showed only marginally significant alterations in the mice treated with TiO_2_/RB@CSNP alone, without irradiation. The administration of TiO_2_/RB@CSNP in the laser, microwave, and laser and microwave combination activated groups'showed significantly necrotic large foci areas (85–90%) in regards to the untreated control DMBA/croton oil-SKCA-induced mice. Additionally, all DMBA/croton oil-SKCA-induced mice treated with IRL, MW, or IRK and MW combination only showed considerable areas of necrosis regarding to the untreated control DMBA/croton oil-SKCA-induced group.

## Discussion

Skin cancer is now the sixth most prevalent malignancy worldwide to be reported, which has an impact on both the economic and global health. The statistics on SKCA have been further worsened by industrialization, genetic manipulation, and the rapidly increasing environmental changes. The cost, toxicity, and bioavailability of several current treatment modalities, including surgery, radiation, conventional chemotherapy and immunotherapy, are causing a loss in their efficacy in treating SKCA and a decrease in patient compliance. To date, there have been a number of Nano-technological developments that have helped to overcome this constraint. Of all the nanomaterial's, nanoparticles have provided enormous benefits by serving as medication carriers and therapeutic agents for the amazing treatment of SKCA. Because of their tiny size and high surface area to volume ratio, targeted therapy with nanoparticles increases the uptake of SKCA through their leaky vasculature, which improves therapeutic efficacy [1, 5, 6, 34–36].

The results of SRB assay is a valuable test that measures viable cell count precisely and evaluates the cytotoxicity of drugs used in cancer treatment. Additionally, one of the most important mechanisms causing cancer is the disruption of the cell cycle that is the basic process which governs a cell's life activities. Moreover, three forms of programmed cell death that counteract biological processes of proliferation and are essential to appropriate development are apoptosis, necrosis, and autophagy. Since most malignancies require tumor cells to have the ability to overpower apoptosis in order to survive, it is thought that the formation and growth of tumors are related to blocked apoptosis and aberrant proliferation [37–40].

The findings of the SRB assay (cytotoxicity) and flow cytometer (cell cycle analysis, apoptosis, necrosis, and autophagy) in this study showed that there was only a minor impact on the SKCA-A-375 cell line following a 24-h incubation period and treatment with TiO_2_/RB@CSNP without activation. The SKCA-A-375 cell line is not significantly affected by the application of laser and shockwave in the absence of TiO_2_/RB@CSNP. The effectiveness of the microwave (MWDT) and the laser (PDT) is increased when TiO_2_/RB@CSNP is present. The obtained results demonstrated that the combined therapeutic method (MWPDT) is more effective than either IRL or MW alone in treating the SKCA-A-375 cell line (cell viability decreased in a dose-responsive manner, the cell cycle progression in G0/G1 was slowed down, and cell death was induced as evidenced by an increase in the population of Pre-G cells, an increase in early and late apoptosis and necrosis, and an increase in autophagic cell death). Our findings are consistent with prior research [41–45]. that demonstrated that the sensitizer TiO_2_/RB@CSNP with both MW (-Micro) and IRL (-photo) combined activation (MWPDT) had the greatest inhibitory effect (cell cycle arrest; progression decline of cell cycle in G0/G1 phase) and destroyed tumor cells (in a dose-dependent manner, reduction cell viability, increasing apoptosis, necrosis, and autophagy). This was followed by TiO_2_/RB@CSNP-MW and IRL activation alone (MWDT and PDT), with MWPDT more effective than MWDT and PDT.

7,12-dimethylbenz[a]-anthracene (DMBA) is a compelling skin carcinogen that increases the occurrence of SKCA, along with the use of SKCA promoters like croton oil. SKCA spreads in three phases: initiation, promotion, and progression. DMBA causes SKCA through various processes, including the formation of dihydrodiol-epoxide (carcinogen) or 1,2-epoxide-3,4-diol DMBA by cytochrome P450 family 1 subfamily A, B member 1 (CYP1 A1, CYP1B1) and cytochrome P450 enzyme (CYP450). This leads to the formation of deoxyribose nucleic acid (DNA) adducts. The ultimate carcinogen that mediates carcinogenesis causes oxidative DNA damage, chronic inflammation, and reactive oxygen species (ROS) overproduction, which in turn causes mutations necessary for tumor formation [46–48].

The current investigation offers proof of the oxidants that the DMBA/croton oil-induced SKCA is advancing. The MDA levels in the untreated DMBA/croton oil-SKCA-induced control mice were significantly higher. Regarding to untreated DMBA/croton oil-SKCA-induced control mice, MDA levels were considerably reduced in all TiO_2_/RB@CSNP treated groups (non irradiated, laser-activated, microwave-activated, and combination of laser and microwave-activated mice). Significant reductions in the TAC backup and in the activities of Catalase, GST, GPx, GR, and SOD further disrupt the antioxidant system. The antioxidant levels in the DMBA/croton oil-SKCA-induced control mice which were not treated were significantly lower. Antioxidant levels were considerably higher in all TiO_2_/RB@CSNP treated groups (laser-activated, microwave-activated, and combination of laser and microwave-activated) than in untreated control DMBA/croton oil-SKCA-induced mice. Previous studies have shown that induction of DMBA/croton oil SKCA results in a rise in MDA significantly and a decline in TAC, and Catalase, GST, GPx, GR, SOD activities [49–54]. It was discovered that mice with SKCA generated by DMBA/croton oil showed reduced activity in scavenging free radicals and were more vulnerable to lipid peroxidation than mice from healthy normal control. As previously mentioned, DMBA/croton oil-SKCA-induction promotes the generation of various free radical metabolites, leading to an excess and accumulation of ROS as well as an increase in the oxidation of cell phospholipid polyunsaturated fatty acid bilayers, which in turn raises MDA levels. The deterioration of enzymatic and non-enzymatic antioxidants is a critical step in the oxidative stress development and cancer evolve. These antioxidants, both enzymatic and non-enzymatic, were continuously depleted, which explained why they were needed to detoxify DMBA/croton oil-SKCA-induced ROS and their metabolites, which explained why they were lacking. The accumulation of ROS may compromise the catalytic activity of enzymatic antioxidants [48–54]. Our findings demonstrated how the anti-lipid peroxidative action of non activated part of TiO_2_/RB@CSNP is supported by its ability to scavenge free radical generation decreasing MDA levels. Furthermore shows the effectiveness of TiO_2_/RB@CSNP as MWPS and its action upon activation by PDT, MWDT, and MWPDT, which eliminated DMBA/croton oil-SKCA cancerous cells; the primary ROS source, causing a restoration and rise in antioxidant enzyme activities and restrict the depletion of antioxidant system and a transition from a cancerous state to a state that is almost normal. Our results agreed with previous studies [55–63] reported the biochemical MWPDT impact (restoration of enzymatic and non-enzymatic antioxidants to near normal levels, reduction of MDA levels, and destruction of DMBA/croton oil-SKCA cells, the fundamental root cause of ROS, and prevention of antioxidant exhaustion by ROS generated from DMBA/croton oil-SKCA-induced cells) was greatest when TiO_2_/RB@CSNP was present as a MWPS in conjunction with both MW (-Micro) and IRL (-photo) activation, followed by TiO_2_/RB@CSNP-MW and IRL activation alone.

In the DMBA/croton oil-SKCA-induced groups in our investigation; there was a substantial increase in ALT and AST, suggesting liver cell damage. Elevated levels of several liver enzymes in serum are associated with cellular leakage of those enzymes into the bloodstream, which is indicative of damage to the hepatocyte's integrity in the cell membrane. Because DMBA/croton oil disrupts metabolism and induces organ failure, the results of the present study are in line with previous studies in showing that hepatic function was reduced in DMBA/croton oil-SKCA-induced animals regarding to control normal mice [46–52, 64–72]. The current study discovered that TiO_2_/RB@CSNP decreased ALT and AST levels, indicating liver protection. Furthermore, this bolsters the protective efficacy of TiO_2_/RB@CSNP in averting hepatic-dysfunction in mice induced by DMBA/croton oil-SKCA. Additionally, DMBA/croton oil triggered a clear elevation in creatinine and urea in the DMBA/croton oil-SKCA-induced groups in this study, indicating renal damage. It has been demonstrated that cardiac and hepatic damage causes renal dysfunction in DMBA/croton oil-SKCA-induced mice, resulting in increased tubular and glomerular congestion. Due to this congestion, results in renal interstitial pressure increase over the entire capillary and tubule system. The findings of this investigation support previous findings by showing that, compared to control normal mice, DMBA/croton oil-SKCA-induced mice had decreased renal function [48–54, 64–72]. The current study discovered that TiO_2_/RB@CSNP decreased serum levels of creatinine and urea, which evoke kidney protection. Moreover, this validates the preventative activity of TiO_2_/RB@CSNP in averting renal impairment in mice that is induced by DMBA/croton oil-SKCA. Our findings demonstrated that the non-activated component of TiO_2_/RB@CSNP scavenges free radicals generated by DMBA/croton oil-SKCA induction, protecting the liver and kidneys. Furthermore, DMBA/croton oil, the primary cause of ROS, is efficiently eliminated by activated TiO_2_/RB@CSNP, which results in the recovery of liver and kidney function as well as transition from a malignant to a condition that is almost normal. The MWPDT was superior to MWDT and PDT, and the ameliorating impact was greatest when TiO_2_/RB@CSNP was present as a MWPS in combination with both MW (-Micro) and IRL (-photo) activation, followed by TiO_2_/RB@CSNP-mw and IRL activation only.

The expressions of TNF alpha, Caspase (3,9), p53, Bcl-2, Bax, and VEGF were molecularly assessed in the current study as indicators of DMBA/croton oil-induced SKCA treatment and inhibition of angiogenesis, respectively. The results indicate a significant negative correlation between the gene expressions and DMBA/croton oil-SKCA-induction, but a positive marked correlation between treatment with different modalities in the presence of TiO_2_/RB@CSNP and the gene expressions. The MWPDT with (TiO_2_/RB@CSNP) treated mice groups had significantly higher expression of the genes TNF alpha, caspase 3, 9, Bax, p53 than did the PDT or MWDT with (TiO_2_/RB@CSNP), IRL or MW only without (TiO_2_/RB@CSNP), and the untreated DMBA/croton oil-SKCA-induced mice. On the other hand, in the untreated DMBA/croton oil-SKCA-induced animals, there was a positive association between Bcl-2 and VEGF expressions, whereas in the TiO_2_/RB@CSNP presence, there was a negative correlation between treatment modalities with VEGF and Bcl-2 expressions. Compared to mice subjected to PDT or MWDT with (TiO_2_/RB@CSNP) alone, and then IRL or MW alone without (TiO_2_/RB@CSNP) alone, the expressions of the VEGF and Bcl-2 genes were considerably lower in mice subjected to MWPDT with (TiO_2_/RB@CSNP). In DMBA/croton oil stimulated SKCA untreated mice group, the expression was highest. Our study's results, which are consistent with those of other studies, show that expressions of TNF alpha, Caspase (3,9), Bax, p53, VEGF and Bcl-2 genes as a reliable indicators of cancer-relevant treatment [32, 72–76]. demonstrating the MWPDT implications on molecular level (VEGF and Bcl-2 genes down regulation; limiting the angiogenesis capabilities and restricting the proliferation of DMBA/croton oil-induced SKCA cells) and (TNF alpha, Bax, p53, and caspase 3, 9 pro-apoptotic genes up regulation; directing DMBA/croton oil-induced SKCA cells to be eradicated by either necrosis or apoptosis and promoting the immune system). Additionally, MWPDT was superior to MWDT and PDT when TiO_2_/RB@CSNP was used as a MWPS in combination with both MW (-Micro) and IRL (-photo) activation.

Current study indicated that TiO_2_/RB@CSNP could be used as a micro- and photo- sensitizer to cure in vivo SKCA caused by DMBA/croton oil. The TiO_2_/RB@CSNP significantly reduces cancer and causes cell death. It can be triggered by chemical activation mechanisms that are mediated by photons or microwave. The biological impact of MWPDT may be elaborated by micro-photochemical/physical mechanisms, such as the excitation of a ground state MWPS (TiO_2_/RB@CSNP) molecule in a singlet state by laser light and microwave, which propels an electron into a higher energy orbital. The MWPS excited triplet state has a comparatively long half-life (~ microseconds) at lower energies until internal or fluorescence conversion returns it to the ground state (~ nanoseconds). On the other hand, the spin of the excited electron inverts. Phosphorescence loses its excitation energy and to the ground singlet state returns in a spin-forbidden transfer. Triplet oxygen excitation can either transfer an electron to superoxide anions, which can produce a variety of ROS that can seriously harm biological systems, or it can transfer its energy to triplet molecular oxygen, which can produce reactive physiologically stimulated singlet state oxygen that can cause serious harm to biological systems. Both the innate and adaptive immune systems respond to MWPDT with a robust defense. Additionally, it kills cells by a variety of mechanisms, including apoptosis, autophagy, and necrosis.

As of this moment, our research indicates that TiO_2_/RB@CSNP may be used as a photo- and microwave sensitizer to cure in vivo SKCA caused by DMBA/croton oil. The TiO_2_/RB@CSNP significantly inhibits tumor growth and causes cell death, which may be triggered by chemical activation processes mediated by photons or microwave. Anticancer effects can be obtained by using microwave and laser in TiO_2_/RB@CSNP presence. It is suggested that light photon dynamic therapy in conjunction with microwave dynamic therapy is a very effective anticancer treatment. Based on our research, TiO_2_/RB@CSNP shows great promise as a novel sensitizer and as a helpful drug delivery system for micro-photodynamic therapy (MWPDT).

## Conclusion

The application of titanium dioxide and rose bengal conjugated chitosan nanoparticles (TiO_2_/RB@CSNP) as a sensitizer delivery system for micro-photodynamic treatment (MWPDT) of skin cancer in vitro (using the A-375 cell line) and in vivo (using DMBA/croton oil-induced mice) demonstrated significant results in this work, indicating promising outcomes in the treatment of cancer. In addition, TiO_2_/RB-CSNP's many benefits, including its low systemic toxicity and excellent bioavailability, make it a feasible alternative for treating cancer. Moreover, the combination of TiO_2_/RB-CSNP@IRL@MW has opened up a wide range of options for anticancer drugs for the effective eradication of skin cancer.

### Recommendation

The current study demonstrated the potential of titanium dioxide and rose bengal conjugated chitosan nanoparticles (TiO_2_/RB@CSNP) as a novel sensitizer in conjunction with micro-photodynamic therapy (MWPDT), a therapeutic approach that is still needs more confirmation and further validation for the treatment of skin cancer. It is strongly advised to conduct additional research using techniques that safely apply this cutting-edge approach and technology to people and monitor modifications in various biophysical and/or biochemical indicators.

## Data Availability

All data generated or analyzed during this study are included in this published article [and its supplementary information files].

## Abbreviations

ALT: Alanine aminotransferase
AST: Aspartate aminotransferase
CAT: Catalase
DMBA: 7,12-Dimethylbenz[a]anthracene
GPx: Glutathione peroxidase
GR: Glutathione reductase
GSH: Reduced glutathione
GST: Glutathione-S-transferase
MDA: Malondialdehyde
MWDT: Microwave-dynamic treatment
MWPDT: Micro-photo-dynamic therapy
PDT: Photodynamic therapy
ROS: Reactive oxygen species
SKCA: Skin cancer
SOD: Superoxide dismutase
SRB: Sulforhodamine B
TAC: Total antioxidant capacity
TiO_2_/RB@CSNP: Titanium Dioxide / Rose Bengal conjugated chitosan nanoparticles
